# Editorial Note: Immuno-PCR - A New Tool for Paleomicrobiology: The Plague Paradigm

**DOI:** 10.1371/journal.pone.0348536

**Published:** 2026-05-04

**Authors:** The PLOS One Editors

The *PLOS One* Editors issue this notice to update the previously published Expression of Concern on this article [1,2].

Following the publication of the article and Expression of Concern [1,2], PLOS investigated concerns pertaining to the reported ethical approval and the article’s adherence to *PLOS One*’s research ethics policies.

Specifically, concerns were raised about insufficient reporting of ethics approval and research permissions for the samples included in this study. The Materials and Methods section of this article reports that the study involved the use of human teeth collected from healthy patients obtaining dental care in Lille and Marseille, France, and ancient teeth collected from archaeological sites in France and Italy. The Ethics statement reads that the study was approved by the Ethic Committee, Institut Fédératif de Recherche 48 (IFR48), Marseilles, France, and that, according to French law, no permission is required for scientific investigations of old mass graves [3].

During editorial review of the information provided in the Materials and Methods section, several questions were raised:

- The article does not report the reference number of the approval granted by the IFR48, and it does not report when these samples were collected. This information is required to determine whether the article is in compliance with PLOS’ Human Subjects Research policy.
- The article does not mention local authorization for the use of ancient teeth samples collected from Venice, Italy. Under article 91 of the Italian Cultural Heritage and Landscape Code, archaeological findings established since 1909 are property of the state, and D.Lgs. 42/2004 of this code mandates the protection and conservation of human remains as part of the nation’s heritage. To PLOS’ understanding, authorization from the regional Soprintendenza Archeologia, Belle Arti e Paesaggio would have been required for the destructive analysis described in the article.

Neither the authors nor the institute responded to PLOS’ request for further clarification and documentation. In the absence of the requested information, PLOS is unable to confirm that this study was conducted in compliance with *PLOS One*’s research ethics policies.

In light of the unresolved issues, the Expression of Concern stands.
